# Forty-nine years in Biomaterials Science: an interview with Buddy Ratner

**DOI:** 10.4155/fsoa-2016-0078

**Published:** 2017-02-06

**Authors:** Buddy Ratner

**Affiliations:** 1 Department of Bioengineering, University of Washington, Seattle, WA 98195, USA; 2 Department of Chemical Engineering, University of Washington, Seattle, WA 98195, USA

**Keywords:** biocompatibility, biomaterials

## Abstract

**Buddy Ratner talks to Francesca Lake, Managing Editor.** After receiving his PhD in polymer chemistry from the Polytechnic Institute of Brooklyn (USA) in 1972, Ratner moved to the University of Washington (USA), where he has since become joint professor of bioengineering and chemical engineering and Michael L & Myrna Darland Endowed Chair in Technology Commercialization. Since 1996, he has led the UWEB Research Center for Biomaterials at the University of Washington, originally funded by the National Science Foundation. A pioneer of the biomaterials field, Ratner's research interests include biomaterials, tissue engineering, polymers, biocompatibility and surface analysis of organic materials. A leader in the field, he has received numerous awards, has launched several companies and holds over 20 patents.

## Q Can you tell us a little more about your current position & what led you to where you are today?

In 1972 I received a PhD in polymer science. I was working on hydrogels for kidney dialysis membranes and had an offer to come to the University of Washington in Seattle for a post-doc working in blood compatibility. Then, Seattle was not a very well-known place, but now everyone knows it owing to some transformational corporate start-ups and a vibrant music scene! That was in 1972, and 44 years later I am still here. From the very first moment I experienced Seattle, I knew this was a place I would like to live. In addition, I enjoyed the environment of the University of Washington, which I found had a very rich intellectual life and a strong interplay between basic sciences and medicine – it seemed like a good place to launch a career. I initially came to work with Professor Allan Hoffman, who offered me a postdoctoral position in 1972, and we worked very closely together for many years until my independent career began taking me in new directions. Along the way, I gradually rose up the academic ranks – I am now a full professor. I started in the Chemical Engineering Department but over the years the Bioengineering Department became more and more significant in its impact and eventually they made me an offer to become part of that department. I am still part of both – Chemical Engineering and Bioengineering – and my students also come from both. I also lead an industry consortium called University of Washington Engineered Biomaterials (UWEB). UWEB, my graduate students, teaching and a number of projects keep me busy and fulfilled. However, I have not taken a major administrative role – I am perfectly pleased with my job as professor. I wanted to be a professor since I was 10 years old, and I have achieved that.

## Q You have had quite an eminent career in biomaterials & engineering – what would you say has been your biggest achievement?

The biggest achievement was when we received funding from the National Science Foundation (NSF) to launch the UWEB center. I will give you more background about UWEB and where its intellectual foundation and impetus came from: I originally came to Seattle to work on blood compatibility of artificial hearts, but also got pretty quickly into the more general subject of biocompatibility along with my colleague Professor Tom Horbett. Tom's background was Biochemistry and mine Polymer Chemistry; so, together we could examine the biology of biocompatibility at the same time as having some sophistication in Material Science. Tom and I proposed a very simple experiment in the mid-1970s in a grant to the NIH; we would study polyethylene and gold, two seemingly simple yet obviously very different (from a materials standpoint), inert materials. These would be implanted in animals and undergo various tests so that we would understand the differences between the two in the way they healed. Launching into these experiments, we found that *in vitro* there were many differences in the way cells and proteins interacted with these materials. One of the surprises when we implanted these materials into animals was that the healing response was essentially indistinguishable between the two, which was referred to as the classic foreign body reaction. Back in that era, we always thought the foreign body reaction was driven by the nature of the materials – it turned out it was not. We continued exploring various aspects but kept coming up with the same results. It seemed very hard to perturb the way the body healed implanted materials. Everything that was inert and not giving off anything toxic healed the same way; the body just put a wall around it. We came to appreciate some of the consequences of that for clinical medicines, where many devices are isolated from the body as opposed to being integrated.

The other interesting thing that was happening at that time was that there were huge advances in cellular and molecular biology – to give an example, when we started out in the early 1970s we did not know that cells had surface receptors; nobody had discovered cell receptors back then or knew that cytokines signaled between cells. In the 1980s there were further tremendous developments that led us to understand much better how cells interacted with their environment. Thus, we were interested in the issue of biocompatibility in healing, and we had all this new information from biology; so, based on this, we put a proposal into the NSF for an engineering research center that would take new developments in biology and enhance or improve the healing or integration of the biomaterials that were at this point being widely used in medical devices. The NSF liked this idea and invested 40 million dollars into the University of Washington and in this program which led to a few classes of materials that instead of being walled-off actually integrated and healed into the body. For example, we could use these new materials on electrodes and could keep electrical communication going; if we used them in drug delivery devices we could prevent encapsulation that blocks the delivery of molecules. There are many other examples of where such integrated healing gives a very different outcome for a medical device. It allows us to do many things that were not possible – we think we can use the same idea (we are working on this right now) for better vascular prostheses.

To get back to your original question, I think the funding of this broad-ranging program, which I directed for 12 years under NSF funding and continue to direct now as an independent program, is one of the hallmark achievements for my career. It gave us the funding to allow us to bring a team of approximately 20 faculty members together to focus very tightly on an important medical problem and out came successful materials that have been translated to humans in a couple of companies.

## Q What else can we expect from UWEB in the near future?

As we speak, we are taking a lot of these ideas forward to launch a new center at the University of Washington – the Centre for Dialysis Innovation. As I mentioned, when I did my PhD work I was working on dialysis membranes – that was in the very early days of kidney dialysis. Throughout most of human history, if someone's kidneys failed, the went into end-stage renal failure, they died an unpleasant death within 3 weeks – there simply were no other options. In the 1930s in Holland, Dr Willem Kolff demonstrated that one could filter a patient's blood and keep them alive. Kolff's work was brilliant and pioneering in demonstrating this. However, the way he achieved access to the bloodstream of these patients was through a fairly major surgical procedure; so, by the time he had connected the patients to his dialysis machine about six times, the patients ran out of sites that they could hook this machine into so they had to let them go. This demonstrated the potential to dialyze humans and keep them alive, but really was not a practical solution to the issue. Jump ahead to the University of Washington in the 1960s, and three individuals came together: an MD nephrologist named Belding Scribner; a bioengineer named Wayne Quinton; and a chemical engineer named Les Babb, who built the first practical artificial kidneys that would allow blood to be dialyzed on a routine basis. That led to the opening of the world's first kidney dialysis centers here in Seattle and the field of dialysis was launched. In the early days, dialysis machines were in very short supply so it was actually important in the field of bioethics too because there were not enough machines for the thousands of people who needed them. We had a group referred to as a ‘hooded committee’ in Seattle that made decisions on who would and would not receive these treatments – a respected clergyman, a mother with three children or a brilliant scientist – who is most deserving of this limited resource? Much thinking in bioethics originated out of the development of the artificial kidney and kidney dialysis.

We now have the situation where there are 400,000 people in the USA, and about 2 million worldwide, receiving dialysis. There is unfortunately a dark side to the story in that when a patient starts kidney dialysis their average lifetime is roughly 3 years; the basic procedure of cleaning the blood works but there are so many complications associated with the procedure that it significantly impacts the lifespan of most people. The other option, kidney transplants, seems like a good option except that there are not nearly enough transplants to go around; so, most patients are still on dialysis. Owing to the grim prognosis for those on dialysis, Professor Jonathan Himmelfarb, an MD nephrologist, and I began to brainstorm a while ago on why these patients were dying and identified a series of complications including things like blood compatibility, blood access, hyperplasia, biofilms and infections, inadequate membranes – a whole series of complications that shorten the lives of patients. We envisioned that instead of addressing each of these one at a time, what if we launched a center that would bring together a team of scientists focused on the problems of kidney dialysis in a systemic manner, with the explicit goal of developing a set of technologies that will revolutionize kidney dialysis in 5 years. At this same time in Seattle, we have had the world's first clinical trial of a wearable artificial kidney – a device that does not tie the patient to a bed three-times a week for 4 h. The device is brilliant but it exacerbates almost every one of these aforementioned problems in dialysis. Again, to address this we are bringing together this team of outstanding scientists from many different areas to see if we can transform the whole area of kidney dialysis. The research work behind this involves blood compatibility and bacterial biofilms, areas of technology I have been involved in extensively; and it involves healing and regeneration, so we are using the UWEB contributions to understanding in that area. I see a good part of my future as being the output from the Centre for Dialysis Innovation, which I hope will be transformational and address many of the pervasive problems with existing kidney dialysis.

## Q What exciting advances should we be looking out for in the next 5 years within the biomaterials arena?

There are a lot of exciting things that are happening. The field of biomaterials has always embraced new ideas and developments and that is still happening. One of the things that I see as being tremendously important in the future is the whole area of 3D printing – it is really becoming quite revolutionary. Many of us have worked extensively in biomaterials, but nobody wants a biomaterial, they want a medical device. I see 3D printing allowing a biomaterials scientist to make devices that can be tested – we can also do new things with materials by combining them. A very closely related area is bioprinting, an idea that came from Professor Thomas Boland. I am very proud to say, Tom was one of my PhD students. Here the option is to actually print tissues using biomaterials and living cells and proteins. I think this is quite transformational.

Another interesting area is a new concept I call ‘cell plasticity’. Through most of Modern Biology we have used the term ‘terminally differentiate’ – a mature cell cannot go any place. Now people are getting the idea that cells are actually very plastic, they change from one cell type to another, and the ability to control these transformations means almost any cell has a stem cell-like ability. We do have defined stem cells but I think the ability to take an adult cell and to crank it back to a more plastic, stem-like state, then direct it, gives us tremendous options in the area we call regenerative medicine. Within the initial biomaterials ideas we said ‘well if a part fails we'll replace it with plastic and metal’ – the regenerative medicine field says ‘if a part fails we'll just regrow it’. Regenerative medicine interest is increasing and efforts in that area are rightly accelerating. The interesting thing is, this still requires an extensive use of biomaterials in many different forms such as scaffolds and drug delivery platforms, so biomaterials are an important foundation technology to make regenerative medicine a reality.

There are also other potentially transformational developments on the horizon, such as the ability to routinely edit the genome of various cell types to allow them to do things that we have not been able to do before. There have been just in the last 3–5 years remarkable developments that continue to add interest, impetus and excitement to the biomaterials field, which has always been flexible to new ideas.

## Q One of your keen interests is in innovation & start-ups. What would you say are the biggest barriers to moving biomaterials work into the clinic at the moment?

Well, if I were developing an application for my smartphone I could write some code and in 2 or 3 months be selling the product on the market to continue to finance my programmers and my staff, allowing me to develop that application. From a biomaterials and medical devices standpoint you have a great idea and then it is 10 years before you get it into the market and into patients, due to regulatory, competitive and commercialization barriers. You, therefore, have a 10-year span with no money coming in. The ability to fund such endeavors takes much ingenuity. A lot of people are doing this, but it is hard work and you are often biting your nails at the possibility of running out of cash, particularly as you get closer to the end point and the need for cash in both product development and clinical testing becomes greater and greater. It is a tricky path one has to walk to get from a smart idea in a laboratory to a commercial product that is benefiting patients and improving medicine. I actually teach two courses at the University of Washington on how one navigates this tricky territory. A lot of people have done it, yet a lot of people have failed doing it. We want to give our students the best possible information and the best possible strategies that will allow them to take their smart ideas to patients.

## Q What top tips would you give someone who was looking to start on that path?

I teach my class with a series of mantras. An important mantra is ‘get the best CEO you can’. An academic scientist may be smart and brilliant, but the ability to get a product on the market takes tremendous focus, and you cannot be worried about students, reading papers, and writing papers and grants at the same time. A CEO tends to be very good at this intensive focus, plus they usually have the financial contacts. We have some good examples of academic scientists who have been CEOs but I think the best examples are where committed CEOs come in and take a leadership role to take the company forward and strategize about what is important to get things on the market. The other mantra, which sounds about a bit callous perhaps, is ‘Cash is King’. When you are in the start-up world everything depends on having the cash to do things. As I mentioned, it is often a tricky proposition to keep the cash coming in for 10 years. However, you have to keep that in the forefront as without cash you do not have employees, and you do not have the resources to develop your product and get it onto the market.

## Q Finally, what are your hopes for the future of biomaterials research? Will we ever reach perfection?

If we go back to the famous Sci-Fi series Star Trek, one of the classic enemies of the Star Trek team were these things called ‘the Borg’, which wanted to subsume humans. They had a machine–biological interface that was totally seamless, that hooked into you and integrated with you. They were quite malicious, but I thought the biomaterial science behind that was brilliant (which of course was done with special effects!). However, this seamless integration between synthetic materials and biology is where much of my research is heading. There are also so many new developments in electronics and sensors, and I can envisage a new class of biomaterials that again seamlessly integrates with humans and gives us a monitoring and healing function, or a restorative function. I see a lot of potential for that future and I am excited by what I like to call ‘seamless integration’!

